# Impact of vitamin A with zinc supplementation on malaria morbidity in Ghana

**DOI:** 10.1186/1475-2891-12-131

**Published:** 2013-09-23

**Authors:** Seth Owusu-Agyei, Sam Newton, Emmanuel Mahama, Lawrence Gyabaa Febir, Martha Ali, Kwame Adjei, Kofi Tchum, Latifa Alhassan, Thabisile Moleah, Sherry A Tanumihardjo

**Affiliations:** 1 Kintampo Health Research Centre (KHRC), Ghana Health Service, P.O.Box 200Kintampo, Brong-Ahafo Region, Ghana; 2 International Atomic Energy Agency, P O Box 100, A-1400, Vienna, Austria; 3 Department of Nutritional Sciences, University of Wisconsin, Madison, USA

**Keywords:** Vitamin A, Zinc, Malaria, Morbidity, MRDR

## Abstract

**Background:**

Malaria is a leading cause of morbidity and mortality among young children and is estimated to cause at least 1 million deaths each year especially among pregnant women and young children under the age of five years. Vitamin A supplementation is known to reduce morbidity and mortality in young children. Zinc is required for growth and immunity and we sought to replicate the study by Zeba et al. which showed 30% lower cases of clinical malaria in children on a combination of zinc and a large dose of vitamin A compared with children on vitamin A alone based on the hypothesis that combined vitamin A and zinc reduced symptomatic malaria compared to vitamin A alone.

**Objectives:**

The primary objective was to determine the effect of vitamin A alone vs. vitamin A and zinc supplements on the incidence of clinical malaria and other anthropometric indices. It also sought to assess the effects on the incidence of anaemia, diarrhoea and pneumonia.

**Methods:**

The study was community-based and 200 children between the ages of 6–24 months were randomised to receive either vitamin A (100,000 IU for infants less than 12 months & 200,000 IU for children greater than 12 months and 10 mg daily zinc in the intervention group or vitamin A and zinc placebo for 6 months in the control group.

**Results:**

The number of children who were diagnosed with uncomplicated malaria in the intervention group was 27% significantly lower compared with the children in the control group (p = 0.03). There were, however, no effects on severe malaria, pneumonia, anaemia and diarrhea.

**Conclusions:**

Our study confirms a significant role of vitamin A and zinc in reducing malaria morbidity.

## Introduction

Malaria is a leading cause of morbidity and mortality among young children and imposes a substantial economic and social burden [1]. It is estimated to cause at least 1 million deaths each year especially among pregnant women and young children under the age of five years. It causes chronic anaemia, impaired growth and delayed development in young children [2].

The health of children is also affected by lack of adequate micronutrients [3] hence home fortification may help improve their micronutrient status [4,5]. Vitamin A supplementation is known to reduce morbidity [6,7] and mortality [8] in children. As vitamin A status of people in developing countries is tenuous, high dose supplements are an effective means to avoid the harmful effects of vitamin A deficiency [9] that results in impaired immune function and an increased risk of death [10]. Malnutrition creates an increased risk of mortality from malaria due to deficiencies of critical micronutrients, such as iron, vitamin A, and zinc [11]. Zinc is required for growth and immunity as well as protein synthesis [12]. Potential consequences of zinc deficiency include compromised immune function, increased rates of serious infectious diseases, and impaired growth [13].

The problem of micronutrient deficiency affects many developing countries such as Ghana. A study in 2008 by Zeba et al. in neighbouring Burkina Faso assessed potential benefits of combining zinc and a large dose of vitamin A. The authors hypothesized that children up to 6 years of age receiving combined vitamin A and zinc supplements will have a lower incidence of symptomatic malaria than similar children receiving vitamin A supplements alone. That study, carried out among children between the ages of 6 and 72 months, demonstrated a significant decrease in the incidence of malaria in the supplemented group compared with the placebo group and a delay in subsequent malaria attacks [14].

Our study sought to investigate whether the findings from Zeba et al. (2008) using the combination of vitamin A and zinc supplements could be further elucidated in younger children between the ages of 6 and 24 months, who are mostly fed weaning foods, to demonstrate if there will be an impact on overall morbidity and/or anthropometric indices. The primary objective of this study was to determine the effect of vitamin A alone vs. vitamin A and zinc supplements on the incidence of clinical malaria. In addition, we determined the effects of vitamin A alone vs. vitamin A and zinc supplements on changes in anthropometric measurements, such as weight and length/height, as well as assessing the effect of the study interventions on the incidence of anemia, diarrhea and pneumonia. The tolerability of the supplements was also assessed. We determined the change in zinc status by measuring plasma zinc concentration using atomic absorption spectrometry before and after supplementation. Finally, the changes in vitamin A status were assessed by using the modified relative dose response (MRDR) test during and at the end of the intervention.

## Methods

### Study site

The study was part of a multi-country, longitudinal, placebo-controlled randomised trial involving four countries in Central (Cameroon) and West Africa (Burkina Faso, Ghana and Mali). In Ghana, the study was conducted in 12 randomly selected communities located in a rural area of the Kintampo North Municipality in the Brong Ahafo Region of Ghana. The Kintampo North Municipality has a resident population of about 80,000; majority of whom have a relatively poor socioeconomic status. Previous studies carried out in the Kintampo area indicate severe vitamin A deficiency with 51% of children under 5 years having serum retinol ≤0.70 μmol/L [15]. Previous anthropometric data indicated a prevalence of stunting of 32% and wasting of 4% among children aged 12 months [16].

### Study design

#### Participants and study interventions

This was a community-based study involving 200 randomly selected infants between the ages of 6 and 24 months who were identified from 12 randomly selected communities using listings generated from the Kintampo Health Demographic Surveillance System (KHDSS) database [17]. A screening form was administered by trained field workers to mothers in the communities to identify eligible children for the study. The child of each consenting mother was issued a study ID card containing identification information to be used by trained field staff to identify the child and replenish their weekly supply of zinc/placebo. The study was double blind and eligible children were randomised to receive 10 mg daily zinc gluconate or zinc placebo (maize powder) for 6 months. Mothers were instructed to mix a capsule of zinc/placebo with a small amount of food to be given to the child. Mothers were not prevented from breastfeeding their infants during the period of supplementation. Where there were at least two eligible children in a household only one child was randomly selected to avoid contamination.

#### Enrolment

**Figure 1:**
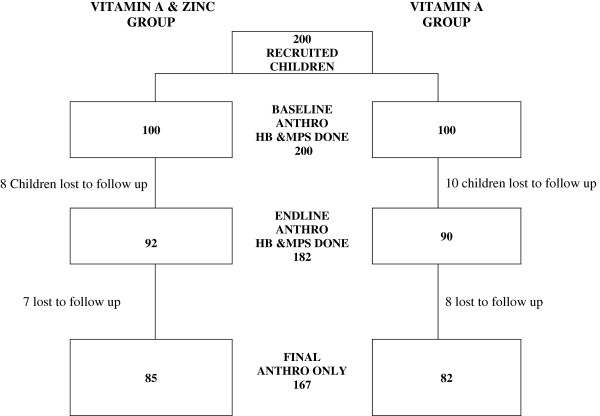
Trial profile of infants recruited into the trial and lost to follow up.

200 infants were recruited and haemoglobin determination was done for all 200 infants at baseline. Blood spots on filter paper were taken from the blood which was collected for malaria parasite assessment for all 200 children. This was followed by vitamin A dosing which started on 30th March 2009. Children less than 12 months of age received 100,000 IU vitamin A and children ≥12 months received 200,000 IU vitamin A at the beginning of the study and at the end of the period of zinc supplementation. After a period of 30 days ± 7 days 3mls of venous blood samples were taken from all available children for vitamin A and zinc analyses. The first week of zinc/placebo supplementation started in May 2009 and continued for 6 months. In all, 18 children were lost to follow up and a second set of 3mls of venous blood samples was collected for the remaining 182 children Figure 1 (92 in the vitamin A and zinc group and 90 in the vitamin A and placebo group) followed by anthropometric measurements. The field work component of the study was successfully completed in November 2009. A third and final anthropometric measurement was carried out 6 months later in May 2010 to assess whether there were any long-term benefits of supplementation with vitamin A and zinc.

### Eligibility

The inclusion of children into the study was based on the child falling in the age group 6 to 24 months, the guardian willing to provide informed consent and the child’s caregiver planning to remain resident in the study area during the study period (at least 7 months). Children were excluded from participation in the study if they were found to have clinical evidence of vitamin A deficiency, severe acute malnutrition, severe illness, child being younger than 6 months or older than 24 months and receipt of vitamin A supplement within the last three months.

### Study procedure

Before the start of fieldwork, chiefs, elders and other members of the selected communities were informed about the objectives of the study. This was followed by community awareness and sensitization meetings in all 12 selected communities about the objectives of the study and the role micronutrients play in the health of children. Recruitment of subjects began on 30th March 2009 after mothers of children had been consented and recruited. At baseline, socio-demographic information was collected; haemoglobin levels were also determined for all study children. All children were supplemented with vitamin A under direct observation by study fieldworkers. A baseline blood sample was collected for determination of vitamin A status using the modified relative dose response (MRDR) test 30 ± 7 days after vitamin A was administered. The test involved administering 5.3 μmol 3,4-didehydroretinyl acetate dissolved in corn oil to the child orally after which blood was taken 4–6 hours later. MRDR values ≥0.060 indicated vitamin A deficiency. Anthropometry (height/length and weight) was also carried out at baseline prior to vitamin A supplementation and at end line following the end of zinc supplementation on 18th October 2009. A second blood draw was carried out for MRDR and zinc analysis at end line after which all participants were dosed with routine vitamin A on completion of the study.

### Morbidity follow-up

Follow up visits were made by fieldworkers to participants to check compliance to the study intervention and to collect data on adverse events and recent illness. Morbidity forms were completed by field workers at the end of each week to collect date on signs and symptoms of illnesses occurring during that week. Data was collected if a child was found to be unwell with fever (temperature ≥37.5°C) or if the mother reported a child’s illness to a field worker, or if there was a history of fever within the past 48 hours. Data was also collected on any other acute illness such as diarrhea, fast breathing or cough in which case the child was then immediately referred to the study clinic and follow up morbidity forms were subsequently completed for participants on days 7, 14 and 28 after hospital referral. Field workers were also taught how to prepare blood slides to determine malaria parasitaemia if the child was found to have a temperature ≥37.5°C or if there was a history of fever within the past 24 hours. Field workers were instructed to put blood drops on filter paper for Polymerase Chain Reaction (PCR) tests and the dried blood spots were kept for future anti malarial drug resistance analysis using genotyping. Haemoglobin measurements were carried out on venous blood which had been collected using an automated HemoCue Hb 201^+^ Blood Hemoglobin photometer, Angelholm, Sweden before and at the end of the study and haemoglobin was regarded as low if it was <10.0 g/dl [18]. The volume of plasma used for analyses of plasma zinc was 0.5 ml and the assessment was carried out at the Institut de Recherche en Sciences de la Santé in Bobo Dioulasso, Burkina Faso using Shimadzu Atomic Absorption Spectrophotometer AA-6300 (P/N 206–51800) Kyoto, Japan.

### Sample size

The primary outcome was incidence of clinical malaria and the sample size was based on the assumption that there will be a reduction of the incidence of clinical malaria of at least 20% among the children who were supplemented with combined vitamin A and zinc compared with those supplemented with vitamin A alone. The significance level was set at p = 0.05 and the power was 80%. Allowing for 10% lost to follow-up, the project required a sample size of 200 children (100 per arm).

### Blood sample analyses

#### Modified relative dose response test

The modified relative dose response (MRDR) test is a biochemical technique used to indirectly determine vitamin A liver reserves in the assessment of vitamin A status in response to intervention trials. The blood samples collected for the MRDR test were stored on ice away from light in a cooler until transported to the laboratory. Clotted blood samples were centrifuged at 600 × *g* for 10 min, and the serum samples were stored at −20°C until shipped. The samples were analyzed for 3, 4-didehydroretinol (DR) and retinol (R) using a standardized method developed specifically for small serum volumes using High Performance Liquid Chromatography (HPLC), JASCO UV-2070 PLUS intelligent UV/VIS Detector, LC-Net II/ADC, PU-2089 PLUS Quaternary Gradient Pump, Tokyo, Japan [19]. As liver vitamin A reserves become depleted, *apo*-retinol binding protein (RBP) accumulates in the liver. For the MRDR test, a challenge dose of 3, 4-didehydroretinyl acetate is administered and the response of dehydroretinol-*holo*-RBP complex is measured in the serum ~5 h after dosing [20]. The MRDR test distinguishes between moderately inadequate and adequate vitamin A status; MRDR values ≤ 0.030 indicate vitamin A adequacy, 0.030 to 0.060 is uncertain vitamin A status, and MRDR ≥0.060 indicate vitamin A deficiency [21].

### Plasma zinc analyses

Standards were run to obtain a calibration curve. The plasma samples were allowed to thaw at room temperature and each sample was gently mixed. 250 μl of the plasma samples was pipetted into 10 ml tubes in duplicates and topped up with 2.5mls n-butanol (6%). The tube was covered with paraffin and vortexed for 3 sec. This was centrifuged for 10 min at 3500 rpm at 21°C. The supernatant was taken out and zinc content was measured. The results were read against the standard calibration curve to obtain the zinc concentration. For quality control purposes controls (Level 1 & 2) were run against the plasma samples [22].

### Data management and statistical analyses

Field supervisors checked all forms manually for completeness and consistency. Forms were double entered in computers, range and consistency checks were performed and discrepancies resolved by reference to the original form using Microsoft visual Foxpro version 9.0 Data Management Software. Clean data were analysed using Stata version 11. Simple descriptive analysis of baseline measures (demographic, socioeconomic, biochemical) was performed across the treatment groups to confirm their comparability. Demographic characteristics of the subjects that were categorical in nature were summarized as proportions while continuous variables were summarized as means. Differences in means of the MRDR tests between intervention and controls were conducted at baseline and end line using the *t* test. Anthropometric indices height-for-age (HA), weight-for-age (WA) and weight-for- height (WH) were expressed as z-scores using the WHO Anthro for personal computers, Version 3.1, 2010: Software for assessing growth and development of children. P < 0.05 was considered significant.

### Ethical issues

Written informed consent was sought from all mothers for participation of their children in the study after a detailed explanation of the purpose, risks and benefits of the study. Mothers who were literate signed the study consent form but finger prints were obtained from mothers who could not sign the consent forms; the thumb printing was done in the presence of a witness. The protocol was approved by the Institutional Ethics Committee of the Kintampo Health Research Centre. Office for Human Research Protections Federal Wide Assurance number 00011103 registration number 0004854. The study was registered with Clinical Trials.gov NCT01782001.

## Results

### Baseline characteristics

A comparison of the baseline data of the intervention and the control groups showed that the two groups were comparable with respect to their socio demographic characteristics. An assessment of availability of amenities, such as electricity and toilets in their homes, showed that the two groups were not significantly different and so was their usage of bed nets. The mean age of enrolled children was similar at baseline and endline (Table 1).

**Table 1:** Comparison of baseline characteristics of mothers of children in the intervention and control groups

	**Intervention group**	**Control group**
	**n = 100 (%)**	**n = 100 (%)**
**Mean age of child (mo)**		
*Baseline (SD*)	14.3 (6.6)	14.8 (6.1)
*End line(SD)*	19.7 (7.8)	20.8 (8.5)
**Sex (%M**)	51 (51.0)	44 (44.0)
**Parity**		
1–3	51 (51.0)	42 (42.0)
4–6	30 (30.0)	33 (33.0)
>6	19 (19.0)	25 (25.0)
**Occupation**		
Farmer/labourer	90 (90.0)	93 (93.0)
Professional	10 (10.0)	7 (7.0)
**Mothers age**		
15–19	33 (33.0)33(33.0)	40 (40.0)
20–29	29 (29.0	37 (37.0)
30–45	5 (5.0))	17 (17.0)
Not known		6 (6.0)
**Highest education level**		
None	54 (54.0)	63 (63.0)
Primary	21 (21.0)	13 (13.0)
Middle	25 (25.0)	24 (24.0)
**Ability to read**		
Yes	26 (26.0)	24 (24.0)
**Bed net use**		
Yes	94 (94.0)	95 (95.0)
**Electricity available**	2 (2.0)	7 (7.0)
**Toilets available**	65 (65.0)	64 (64.0)
**Drinking water**		
Communal tap	66 (66.0)	66 (66.0)
Ground well	6 (6.0)	6 (6.0)
Stream	28 (28.0)	28 (28.0)

No significant difference was noted among treatment groups for all characteristics.

### Anthropometry and haematological indices

There was no significant difference in anthropometric indices between the intervention and control groups at baseline and end line and when this was assessed a further 6 months later, there was still no significant difference. Anaemia rates were not significantly different at baseline and endline between the intervention and the control groups. Plasma zinc levels were as expected higher at end line in the intervention group compared to the control group but this was not statistically different. They were fewer children in the intervention group (23.9%) who were anaemic at end line compared to infants in the control (29.4%) but this was not statistically significant (p = 0.69) (Table 2).

**Table 2:** Infant anthropometric, vitamin A status, haematologic and anaemia indexes at baseline and end of study

	**Intervention (n = 100)**	**Control (n = 100)**	**p**
***Baseline anthro.***			
Child weight (kg)	8.6 (8.3, 8.9)	8.7 (8.4, 9.1)	0.68
Child height (cm)	74.4 (73.1, 75.8)	75.1 (73.8, 76.3)	0.51
Height for age^a^	−0.95 ± 1.01 (−1.15, -0.75)	−1.0 ± −0.96 (−1.22, -0.84)	0.57
Weight for age^a^	−1.1 ± 0.92 (−1.27, -0.89)	−1.1 ± 1.0 (−1.3, -0.89)	0.90
Weight for height^a^	−0.81 ± 0.80 (−0.97, -0.64)	−0.8 ± 1.1 (−0.96, -0.53)	0.67
***Endline anthro.***			
Child weight(kg)	10.3 (9.3, 11.3)	10.4 (9.4, 11.5)	0.88
Child height (cm)	89.6 (69.0, 78.2)	79.9 (78.6, 81.1)	0.34
Height for age^a^	−1.4 ± 1.41 (−1.68, -1.08)	−1.3 ± 1.50 (−1.70, -1.10)	0.95
Weight for age^a^	−0.83 ± 3.13 (−1.49, -0.16)	−0.76 ± 3.18 (−1.42, -0.10)	0.87
Weight for height^a^	−0.16 ± 4.26 (−1.10, -0.74)	−0.10 ± 4.37 (−0.98, -0.83)	0.90
***Final anthro.***			
Child weight (kg)	11.2 (10.8, 11.6)	11.2 (10.8, 11.6)	0.94
Child Height (cm)	85.0 (83.8, 86.1)	84.8 (83.4, 86.2)	0.84
Height for age^a^	0.86 ± 1.33 (0.56, 1.16)	1.19 ± 2.48 (0.65, 1.72)	0.31
Weight for age^a^	0.34 ± 0.37 (−0.40, 1.08)	0.54 ± 0.41 (−0.27, 1.35)	0.72
Weight for height^a^	−0.05 ± 0.48 (−1.01, 0.92)	−0.13 ± 0.45 (−1.02, 0.76)	0.90
***Baseline indices***			
Haem (g/dL)	10.7 (10.4,11.0)	10.8 (10.5, 11.0)	0.84
Anaemia *(n%)*^b^	28/100 (28.0)	31/100 (31)	0.64
Low Vit. A status^y^	59/64 (92.2)	42/47 (89.4)	0.61
Adequate Vit A status^χ^	1/64 (1.6)	0/47 (1.6)	0.39
Plasma zinc(μg/dl)	81 (72, 90)	69 (57, 81)	0.10
***End line indices***			
Haem. (g/dL)	10.9 (10.5, 11.2)	10.8 (10.5, 11.2)	0.93
Anaemia (n%)^b^	21/88 (23.9%)	27/92 (29.4%)	0.69
Low vit. A status^y^	45/57 (78.9)	37/43 (86.1)	0.36
Adequate vit A status^χ^	1/57 (1.8)	1/43 (2.3)	0.84
Received vit A in 6 months	96/100 (96.0)	95/100 (95.0)	0.50
Plasma zinc (μg/dl)	63 (52, 73)	58 (49, 66)	0.48

a- χ ± SD; range in parenthesis (all such values).

b. Defined as haemoglobin concentration <10 g/dl.

y-low vitamin A status MRDR ≥ 0.06.

χ-Adequate vitamin A status MRDR ≤ 0.03.

### Morbidity assessments and malaria parasitaemia

A number of childhood morbidities were assessed during weekly visits from the start of supplementation until the completion of the study which was the week 22-visit. There was no significant difference between children in the intervention and the control groups in any of the conditions assessed during the visits. Geometric mean parasites densities were compared at baseline and end line and the intervention and the control groups did not differ significantly at baseline. However, at end line it was observed that there were consistently lower geometric mean parasite densities in the intervention group compared to the control group for counts greater than or equal to 2000 even though this was not statistically significant; though the numbers of participants were very small, counts between 5,000 and 10,000 were statistically significant (p = 0.02) (Table 3).

**Table 3:** Comparison of geometric mean malaria parasite densities at baseline and end line

**Parasite count**	**Observations**		**Parasite densities**	**(95%CI)**	**p**
	**Intervention**	**Control**	**Intervention**	**Control**	
**Baseline**^**y**^					
<2000	17	15	491 (288,838)	542 (310,949)	0.78
≥2000& < 5000	7	8	2732 (2196,3381)	3285 (2839,3802)	0.10
≥5000& < 10000	11	6	7473 (6488,8608)	6517 (5085,8351)	0.23
≥10000	12	19	25263 (16019,39842)	33042 (22825,47831)	0.33
**Total**	47	48	3281 (1956,5501)	5084 (2932,8811)	0.24
**Endline**^**X**^					
<2000	8	7	576 (245,1354)	390 (146,1037)	0.48
≥2000& < 5000	4	5	2495 (2053,3547)	3175 (2428,4154)	0.08
≥5000& < 10000	4	3	6973 (5856,8303)	8863 (7238,10854)	**0.02**
≥10000	3	7	24485 (6625,90491)	33739 (18049,63068)	0.49
**Total**	19	22	2397 (1119, 5135)	3976 (1659,9257)	0.37

Y 105 out of 200 children had no malaria parasites at baseline.

X-159 out of 182 children who completed follow up had no malaria parasites at end line.

### Clinical malaria

The number of children who were diagnosed with uncomplicated malaria in the intervention group was 27%, which was significantly lower than the children in the control group p = 0.03. However, all other clinical conditions assessed such as severe malaria, pneumonia, anaemia and diarrhoea were not found to be statistically significant between the intervention and the control groups (Table 4).

**Table 4:** Impact of supplementation on malaria and general morbidity

**Morbidity**	**Intervention (87)**	**Control (88)**	**RR (95%CI)**	**p**
	**n (%)**	**n (%)**		
Uncomplicated Malaria	40 (46.0)	55 (62.5)	0.73 (0.55, 0.97)	0.03
Severe malaria	1 (1.1)	1 (1.1)	0.99 (0.06, 15.9)	0.99
Non severe pneumonia	3 (3.4)	1 (1.1)	3.03 (0.32,28.6)	0.33
Severe pneumonia	0	1 (1.1)	**-**	**-**
Acute diarrhoea	9 (10.3)	7 (7.9)	1.30 (0.51,3.33)	0.58
Bloody diarrhoea	7 (8.0)	4 (4.5)	1.77 (0.54,5.83)	0.34

## Discussion

We demonstrated in this randomised controlled trial that giving a combination of vitamin A and zinc supplements to children aged 6–24 months led to 27% significantly fewer cases of clinical attacks compared to children who were given vitamin A alone. This finding suggests a role of zinc in reducing malaria morbidity when given in addition to vitamin A; thus confirming the findings of Zeba et al.^14^ which demonstrated 30% fewer cases of clinical attacks.

Shankar et al. [23] demonstrated that vitamin A supplementation of children 6 months to 5 years of age in Papua New Guinea resulted in decreased malaria parasite densities, severity of infection and febrile episodes, which we also observed in our study. Several cross-sectional studies have shown a relationship between low zinc status and increased incidence of malaria [24,25]. Shankar et al. [26] and Bates et al. [27] have in a pooled analysis demonstrated a 36% (95%CI:9-55%) reduction in the incidence of clinical malaria in children given daily doses of zinc supplements. However in a study in Gambian children by Muller et al. [28], there was no significant difference in the incidence of clinical malaria between the daily zinc and placebo groups. Other studies in Ghana by Binka et al. have not shown a significant effect of vitamin A on malaria parasitaemia rates or parasite densities [29]. We observed that at end line a lower percentage of infants in the intervention group had high malaria parasite counts compared to infants in the control group and it is therefore not surprising that infants in the intervention group had a lower incidence of clinical malaria compared to infants in the control group Table 3.

Our study also showed a modest effect of vitamin and zinc supplementation on weight and length/height gain of the children involved in the study but this was not statistically significant and the reasons for this are not clear. This finding is also similar to results obtained by Rahman et al. [30] in Bangladeshi children between the ages of 12–35 months. The authors concluded that combined short-term zinc supplementation and a single dose of vitamin A had no significant change on weight and length increments in children over a 6-month period. Studies in Danish infants have shown an increase in anthropometric indices between the ages of 6–9 months among infants supplemented with zinc [31] but two other studies showed that low zinc intake did not impair growth [32,33]. We did not find any significant differences in the anthropometric indices of those who were supplemented with zinc and the reasons for this are not too clear.

Humans have no body store for zinc, and bioavailable zinc must be supplied on a regular basis [34]. Zinc deficiency in infancy and early childhood in developing countries leads to stunting [35], infectious disease morbidity [36] and mortality [37] especially from diarrhoea and pneumonia.

Pooled analyses of four randomised controlled trials by the Zinc Collaborative Group showed a 41% reduction in the incidence of pneumonia [38]. In a study in Bangladesh assessing the interaction of the combination of vitamin A and zinc in the prevention of acute lower respiratory infections (ALRI), there was an increased relative risk of ALRI in the zinc supplemented children (RR 1.06, 95% CI 1.01-2.25) when compared to placebo [39] but we are unable to confirm or deny these finding because the number of respiratory diseases reported in our study were low.

There is undisputed evidence of the efficacy of zinc for the treatment of childhood diarrhoea and this has been confirmed by the results of pooled analyses of seven studies in children under the age of five years^38^. Gupta et al. supplemented children 6–41 months of age with 10 mg zinc five times a week and it was observed that children who received this had significantly lower rates of diarrhoea during the period of supplementation [40]; however, our study did not demonstrate any significant difference in the incidence of diarrhoea between the intervention and control groups.

We observed a modest increase in plasma zinc levels from baseline to end line, but this was not statistically significant. Similar findings have been described by Lo et al. [41] who found that plasma zinc concentration increased in children who received daily zinc supplementation for but not in those who received zinc with complementary foods suggesting the role of inhibitors in absorption.

Our study was conducted in an area where the mothers were of low socioeconomic status. More than 90% of children received a dose of vitamin A in the past 6 months. It is important to note that even though the children had been given vitamin A, vitamin A analyses showed that most of them still remained vitamin A deficient Table 2.

A limitation of the study was that the fact that the intervention was self administered, the weekly visits may not have adequately captured compliance to the intervention and the study was powered to look at the incidence of clinical malaria and so may not have been adequately powered to detect the effect of supplementation on other indices.

Our study showed that it was safe to administer vitamin A and zinc to infants as there were no reports of adverse events associated with administering zinc and vitamin A supplements, findings that have been confirmed by Walker and Black [42].

## Conclusions

This study revealed the critical role played by micronutrients, such as vitamin A and zinc, in the reduction of clinical attacks of malaria in young children. Malaria remains a leading cause of morbidity and mortality among young children in developing countries and imposes a huge economic and social burden and any methods that can be used to reduce this burden should be explored.

## Abbreviations

AAS: Atomic absorption spectroscopy; HPLC: High performance liquid chromatography; PCR: Polymerase chain reaction; VA: Vitamin A; VAD: Vitamin A deficiency.

## Competing interests

The authors declare that they have no competing interests.

## Authors’ contribution

SOA, SN, TM and ST made primary contributions to the overall development, rationale, and design of the trial as well as writing the report. SOA, SN, KA, MA and LG coordinated the trial. SOA, SN and EM contributed to data management and analysis. KT, LA and ST conducted and managed the laboratory analyses. SOA, SN and EM conducted the statistical analyses. All authors read and approved the final manuscript.
